# {μ-6,6′-Dimeth­oxy-2,2′-[ethane-1,2-diylbis(nitrilo­methyl­idyne)]diphenolato-1κ^4^ *O* ^6^,*O* ^1^,*O* ^1′^,*O* ^6′^:2κ^4^ *O* ^1^,*N*,*N*′,*O* ^1′^}(methanol-1κ*O*)(perchlorato-1κ*O*)copper(II)sodium(I)

**DOI:** 10.1107/S1600536809010356

**Published:** 2009-03-25

**Authors:** Hui-Quan Xiao, Hua Zhang

**Affiliations:** a Department of Chemistry, Shaoxing University, Shaoxing 312000, People’s Republic of China; b College of Chemical Engineering and Materials, Heilongjiang University, Haerbin 150080, People’s Republic of China

## Abstract

The mol­ecule of the title compound, [CuNa(C_18_H_18_N_2_O_4_)(ClO_4_)(CH_3_OH)], is almost planar  with mean deviation of 5.2 (8)°. The coordination environment of the Cu^II^ ion is distorted square-planar and it is N_2_O_2_-chelated by the 6,6′-dimeth­oxy-2,2′-[ethane-1,2-diylbis(nitrilo­methyl­idyne)]diphenolate Schiff base ligand. The Na atom is chelated by the four O atoms of the Schiff base ligand, and coordinated by a methanol mol­ecule and a perchlorate anion. The six-coordinate Na atom adopts a distorted octa­hedral coordination geometry. The O atoms of the perchlorate anion are disordered over two sites with site-occupancy factors of 0.697 (5) and 0.303 (5). O—H⋯O hydrogen bonding occurs.

## Related literature

For chemical background, see: Lindoy *et al.* (1976 ▶). For related structures, see: Correia *et al.* (2005 ▶). For bond-length data, see: Allen *et al.* (1987 ▶).
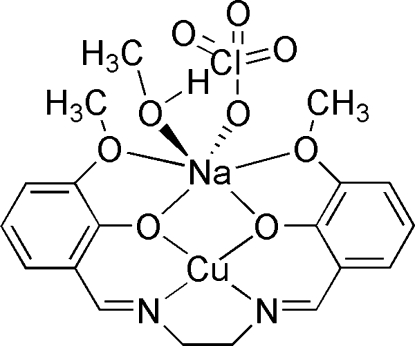


## Experimental

### 

#### Crystal data

[CuNa(C_18_H_18_N_2_O_4_)(ClO_4_)(CH_4_O)]
*M* *_r_* = 544.38
Monoclinic, 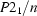

*a* = 12.030 (2) Å
*b* = 8.1444 (14) Å
*c* = 23.381 (4) Å
β = 104.273 (3)°
*V* = 2220.1 (7) Å^3^
*Z* = 4
Mo *K*α radiation
μ = 1.18 mm^−1^
*T* = 273 K
0.17 × 0.15 × 0.11 mm

#### Data collection

Bruker APEXII CCD area-detector diffractometer
Absorption correction: multi-scan (*SADABS*; Sheldrick, 2003 ▶) *T*_min_ = 0.825, *T*_max_ = 0.881
10424 measured reflections
3843 independent reflections
2651 reflections with *I* > 2σ(*I*)
*R*_int_ = 0.039

#### Refinement

*R*[*F* ^2^ > 2σ(*F* ^2^)] = 0.048
*wR*(*F* ^2^) = 0.131
*S* = 1.03
3843 reflections
339 parameters
148 restraints
H-atom parameters constrained
Δρ_max_ = 0.56 e Å^−3^
Δρ_min_ = −0.68 e Å^−3^

### 

Data collection: *APEX2* (Bruker, 2004 ▶); cell refinement: *SAINT-Plus* (Bruker, 2001 ▶); data reduction: *SAINT-Plus*; program(s) used to solve structure: *SHELXS* (Sheldrick, 2008 ▶); program(s) used to refine structure: *SHELXL97* (Sheldrick, 2008 ▶); molecular graphics: *XP* in *SHELXTL* (Sheldrick, 2008 ▶); software used to prepare material for publication: *XP* in *SHELXTL*.

## Supplementary Material

Crystal structure: contains datablocks I, global. DOI: 10.1107/S1600536809010356/pv2143sup1.cif

Structure factors: contains datablocks I. DOI: 10.1107/S1600536809010356/pv2143Isup2.hkl

Additional supplementary materials:  crystallographic information; 3D view; checkCIF report

## Figures and Tables

**Table 1:** Hydrogen-bond geometry (Å, °)

*D*—H⋯*A*	*D*—H	H⋯*A*	*D*⋯*A*	*D*—H⋯*A*
O9—H9*A*⋯O7^i^	0.82	2.11	2.912 (8)	164
O9—H9*A*⋯O5′^i^	0.82	1.99	2.761 (13)	156
O9—H9*A*⋯O5′^i^	0.82	1.99	2.761 (13)	156
O9—H9*A*⋯O7^i^	0.82	2.11	2.912 (8)	164

Symmetry code: (i) 
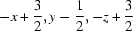
.

**Fig. 1.**
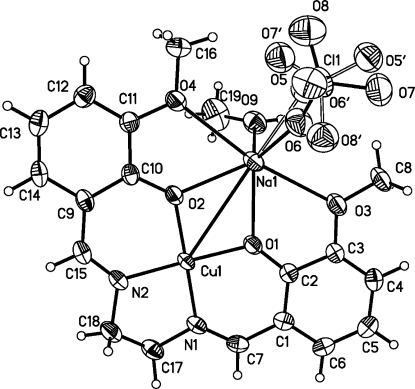
The molecular structure of the title compound, showing 30% probability displacement ellipsoids and the atom-numbering scheme.

## supplementary crystallographic information

### Comment

The schiff bases have been known as effective ligands for metal ions and used in the mechanism of many biochemical processes (Lindoy *et al.*, 1976). *N*,*N*-Disalicylideneethylenediamine type schiff bases present versatile steric, electronic and lipophilic properties. The synthesis, characterization and solution studies of *N*,*N*'-disalicylideneethylenediamine type complexes have been reported recently (Correia *et al.*, 2005). We report here the synthesis and crystal structure of the title compound, (I). The molecular structure of (I) is shown in Fig. 1. The values of the geometric parameters in (I) are normal (Allen *et al.*, 1987). Cu(II) and Na(I) are connected *via* two bridging oxygen atoms of the ligand. The coordination environment of the Cu^2+^ ion is distorted square-planar and it is coordinated by N_2_O_2_ of the Schiff base ligand. The Na atom is chelated by the four O atoms of the 6,6'-dimethoxy-2,2'-(ethane-1,2-diyldiiminodimethylene)diphenol ligand, a methanol and a perchlorate anion. The six-coordinate Na atom adopts a distorted octahedral coordination geometry.

### Experimental

A mixture of 6,6'-dimethoxy-2,2'-(ethane-1,2-diyldiiminodimethylene)diphenol (1 mmol) and copper chloride (1 mmol) in absolute ethanol (15 ml) was stirred for 30 min and sodium perchlorate (1 mmol) was added, stirred for another 15 min and then filtered. The resulting clear orange solution was allowed to evaporate at room temperature for 7 days, after which large orange block-shaped crystals of the title complex suitable for X-ray diffraction analysis were obtained.

### Refinement

The H atoms were fixed geometrically and were treated as riding on their parent atoms, with C—H distances 0.93, 0.96 and 0.97 Å for aryl, methyl and methylene type H-atoms, respectively, and O—H = 0.85 Å. The H-atoms were allowed *U*_iso_(H) = 1.2*U*_eq_(parent aryl and methylene C atoms), or *U*_iso_(H) = 1.5*U*_eq_(methyl C and hydroxyl O atoms). The O-atoms of the perchlorate anion are disordered over two sites with unequal site occupancy factors 0.697 (5) and 0.303 (5).

### Crystal data

**Table:**

[CuNa(C_18_H_18_N_2_O_4_)(ClO_4_)(CH_4_O)]	*F*(000) = 1116
*M**_r_* = 544.38	*D*_x_ = 1.629 Mg m^−^^3^
Monoclinic, *P*2_1_/*n*	Mo *K*α radiation, λ = 0.71073 Å
Hall symbol: -P 2yn	Cell parameters from 2083 reflections
*a* = 12.030 (2) Å	θ = 2.7–21.2°
*b* = 8.1444 (14) Å	µ = 1.18 mm^−^^1^
*c* = 23.381 (4) Å	*T* = 273 K
β = 104.273 (3)°	Block, blue
*V* = 2220.1 (7) Å^3^	0.17 × 0.15 × 0.11 mm
*Z* = 4	

### Data collection

**Table:**

Bruker APEXII CCD area-detector diffractometer	3843 independent reflections
Radiation source: fine-focus sealed tube	2651 reflections with *I* > 2σ(*I*)
graphite	*R*_int_ = 0.039
φ and ω scans	θ_max_ = 25.0°, θ_min_ = 2.2°
Absorption correction: multi-scan (*SADABS*; Sheldrick, 2003)	*h* = −14→10
*T*_min_ = 0.825, *T*_max_ = 0.881	*k* = −9→9
10424 measured reflections	*l* = −27→26

### Refinement

**Table:**

Refinement on *F*^2^	Primary atom site location: structure-invariant direct methods
Least-squares matrix: full	Secondary atom site location: difference Fourier map
*R*[*F*^2^ > 2σ(*F*^2^)] = 0.048	Hydrogen site location: inferred from neighbouring sites
*wR*(*F*^2^) = 0.131	H-atom parameters constrained
*S* = 1.03	*w* = 1/[σ^2^(*F*_o_^2^) + (0.0701*P*)^2^ + 0.3025*P*] where *P* = (*F*_o_^2^ + 2*F*_c_^2^)/3
3843 reflections	(Δ/σ)_max_ = 0.002
339 parameters	Δρ_max_ = 0.56 e Å^−^^3^
148 restraints	Δρ_min_ = −0.68 e Å^−^^3^

### Special details

**Table:**

Geometry. All e.s.d.'s (except the e.s.d. in the dihedral angle between two l.s. planes) are estimated using the full covariance matrix. The cell e.s.d.'s are taken into account individually in the estimation of e.s.d.'s in distances, angles and torsion angles; correlations between e.s.d.'s in cell parameters are only used when they are defined by crystal symmetry. An approximate (isotropic) treatment of cell e.s.d.'s is used for estimating e.s.d.'s involving l.s. planes.
Refinement. Refinement of *F*^2^ against ALL reflections. The weighted *R*-factor *wR* and goodness of fit *S* are based on *F*^2^, conventional *R*-factors *R* are based on *F*, with *F* set to zero for negative *F*^2^. The threshold expression of *F*^2^ > σ(*F*^2^) is used only for calculating *R*-factors(gt) *etc*. and is not relevant to the choice of reflections for refinement. *R*-factors based on *F*^2^ are statistically about twice as large as those based on *F*, and *R*- factors based on ALL data will be even larger.

### Fractional atomic coordinates and isotropic or equivalent isotropic displacement parameters (Å^2^)

**Table:**

	*x*	*y*	*z*	*U*_iso_*/*U*_eq_	Occ. (<1)
Cu1	0.67526 (4)	0.41736 (7)	1.00850 (2)	0.0401 (2)	
Cl1	0.50250 (12)	0.6363 (2)	0.73548 (7)	0.0739 (4)	
Na1	0.64991 (14)	0.4180 (2)	0.86447 (7)	0.0487 (5)	
O1	0.7475 (2)	0.5185 (4)	0.95491 (12)	0.0450 (7)	
O2	0.5730 (2)	0.3261 (4)	0.94164 (12)	0.0469 (8)	
O3	0.8214 (3)	0.6120 (4)	0.86695 (14)	0.0586 (9)	
O4	0.4620 (3)	0.2267 (4)	0.83993 (13)	0.0551 (8)	
O5	0.4054 (5)	0.7314 (9)	0.7368 (3)	0.0941 (18)	0.697 (5)
O6	0.5622 (6)	0.5794 (11)	0.7907 (3)	0.107 (2)	0.697 (5)
O7	0.5660 (5)	0.7560 (8)	0.7127 (3)	0.1002 (19)	0.697 (5)
O8	0.4583 (6)	0.5066 (8)	0.6955 (3)	0.121 (2)	0.697 (5)
O5'	0.5499 (13)	0.648 (2)	0.6860 (5)	0.102 (3)	0.303 (5)
O6'	0.5809 (11)	0.5076 (15)	0.7574 (8)	0.095 (3)	0.303 (5)
O7'	0.3899 (7)	0.596 (2)	0.7381 (8)	0.106 (3)	0.303 (5)
O8'	0.5499 (14)	0.683 (2)	0.7956 (3)	0.100 (3)	0.303 (5)
O9	0.7367 (4)	0.1937 (5)	0.8355 (2)	0.0792 (12)	
H9A	0.7905	0.1921	0.8198	0.119*	
N1	0.7854 (3)	0.4916 (5)	1.07680 (15)	0.0450 (9)	
N2	0.5934 (3)	0.3323 (5)	1.06299 (15)	0.0461 (9)	
C1	0.9026 (4)	0.6473 (5)	1.0254 (2)	0.0438 (11)	
C2	0.8414 (3)	0.6065 (5)	0.9680 (2)	0.0400 (10)	
C3	0.8843 (4)	0.6644 (5)	0.92130 (19)	0.0433 (11)	
C4	0.9803 (4)	0.7612 (6)	0.9308 (2)	0.0533 (12)	
H4	1.0059	0.7997	0.8988	0.064*	
C5	1.0398 (4)	0.8024 (6)	0.9880 (3)	0.0583 (13)	
H5	1.1046	0.8689	0.9944	0.070*	
C6	1.0018 (4)	0.7438 (6)	1.0342 (2)	0.0549 (12)	
H6	1.0424	0.7684	1.0725	0.066*	
C7	0.8704 (4)	0.5871 (6)	1.0767 (2)	0.0476 (11)	
H7	0.9152	0.6201	1.1132	0.057*	
C8	0.8646 (5)	0.6465 (8)	0.8172 (2)	0.0694 (15)	
H8A	0.8684	0.7632	0.8122	0.104*	
H8B	0.8147	0.5994	0.7826	0.104*	
H8C	0.9399	0.6003	0.8229	0.104*	
C9	0.4421 (4)	0.1995 (5)	0.9915 (2)	0.0449 (11)	
C10	0.4820 (4)	0.2381 (5)	0.94127 (19)	0.0404 (10)	
C11	0.4171 (4)	0.1799 (6)	0.8863 (2)	0.0455 (11)	
C12	0.3175 (4)	0.0908 (6)	0.8808 (2)	0.0559 (13)	
H12	0.2759	0.0549	0.8440	0.067*	
C13	0.2806 (4)	0.0556 (6)	0.9310 (3)	0.0639 (15)	
H13	0.2136	−0.0043	0.9278	0.077*	
C14	0.3411 (4)	0.1075 (6)	0.9848 (2)	0.0545 (13)	
H14	0.3151	0.0814	1.0180	0.065*	
C15	0.5014 (4)	0.2496 (6)	1.0498 (2)	0.0501 (12)	
H15	0.4695	0.2188	1.0806	0.060*	
C16	0.4046 (5)	0.1701 (8)	0.7830 (2)	0.0810 (18)	
H16A	0.4001	0.0525	0.7833	0.122*	
H16B	0.4462	0.2042	0.7549	0.122*	
H16C	0.3286	0.2154	0.7723	0.122*	
C17	0.7679 (5)	0.4219 (7)	1.1319 (2)	0.0614 (14)	
H17A	0.7920	0.5000	1.1639	0.074*	
H17B	0.8132	0.3227	1.1420	0.074*	
C18	0.6426 (5)	0.3831 (7)	1.1231 (2)	0.0619 (14)	
H18A	0.6334	0.2961	1.1499	0.074*	
H18B	0.6026	0.4795	1.1320	0.074*	
C19	0.7189 (7)	0.0396 (9)	0.8542 (3)	0.107 (2)	
H19A	0.6615	0.0434	0.8763	0.161*	
H19B	0.7892	−0.0022	0.8788	0.161*	
H19C	0.6935	−0.0309	0.8206	0.161*	

### Atomic displacement parameters (Å^2^)

**Table:**

	*U*^11^	*U*^22^	*U*^33^	*U*^12^	*U*^13^	*U*^23^
Cu1	0.0401 (3)	0.0532 (4)	0.0286 (3)	0.0046 (2)	0.0112 (2)	0.0037 (2)
Cl1	0.0659 (9)	0.0907 (10)	0.0711 (9)	0.0173 (7)	0.0283 (7)	0.0296 (8)
Na1	0.0491 (10)	0.0658 (12)	0.0315 (9)	0.0029 (8)	0.0108 (8)	0.0001 (8)
O1	0.0392 (17)	0.066 (2)	0.0297 (16)	−0.0046 (15)	0.0077 (13)	0.0015 (14)
O2	0.0442 (18)	0.067 (2)	0.0328 (17)	−0.0092 (15)	0.0147 (14)	0.0002 (14)
O3	0.0522 (19)	0.089 (3)	0.0370 (19)	−0.0065 (17)	0.0157 (15)	0.0078 (17)
O4	0.0504 (19)	0.079 (2)	0.0386 (18)	−0.0120 (16)	0.0158 (15)	−0.0082 (16)
O5	0.082 (3)	0.100 (3)	0.099 (3)	0.023 (3)	0.022 (3)	0.000 (3)
O6	0.103 (3)	0.110 (3)	0.099 (3)	0.015 (3)	0.011 (3)	0.029 (3)
O7	0.100 (3)	0.105 (3)	0.102 (3)	−0.007 (3)	0.038 (3)	0.024 (3)
O8	0.122 (3)	0.114 (3)	0.119 (3)	0.012 (3)	0.014 (3)	−0.007 (3)
O5'	0.101 (4)	0.110 (4)	0.099 (4)	0.005 (3)	0.030 (3)	0.008 (3)
O6'	0.094 (4)	0.096 (4)	0.093 (4)	0.009 (4)	0.019 (4)	0.013 (4)
O7'	0.098 (4)	0.114 (4)	0.105 (4)	0.006 (3)	0.022 (3)	0.008 (4)
O8'	0.096 (4)	0.101 (4)	0.101 (4)	0.007 (4)	0.018 (4)	0.016 (4)
O9	0.084 (3)	0.082 (3)	0.080 (3)	0.017 (2)	0.038 (2)	−0.005 (2)
N1	0.052 (2)	0.057 (2)	0.0242 (19)	0.010 (2)	0.0073 (16)	0.0034 (17)
N2	0.055 (2)	0.053 (2)	0.033 (2)	0.0042 (19)	0.0157 (18)	0.0058 (17)
C1	0.044 (3)	0.044 (3)	0.042 (3)	0.008 (2)	0.007 (2)	−0.004 (2)
C2	0.033 (2)	0.044 (3)	0.044 (3)	0.0067 (19)	0.0110 (19)	0.000 (2)
C3	0.042 (3)	0.048 (3)	0.042 (3)	0.008 (2)	0.015 (2)	0.007 (2)
C4	0.052 (3)	0.051 (3)	0.063 (3)	0.001 (2)	0.023 (2)	0.009 (2)
C5	0.055 (3)	0.046 (3)	0.073 (4)	−0.007 (2)	0.014 (3)	−0.004 (3)
C6	0.050 (3)	0.050 (3)	0.061 (3)	−0.003 (2)	0.006 (2)	−0.011 (2)
C7	0.048 (3)	0.052 (3)	0.039 (3)	0.009 (2)	0.002 (2)	−0.004 (2)
C8	0.073 (4)	0.098 (4)	0.043 (3)	0.000 (3)	0.025 (3)	0.015 (3)
C9	0.047 (3)	0.046 (3)	0.047 (3)	0.012 (2)	0.023 (2)	0.009 (2)
C10	0.038 (2)	0.042 (3)	0.043 (3)	0.0073 (19)	0.015 (2)	0.004 (2)
C11	0.042 (3)	0.049 (3)	0.049 (3)	0.002 (2)	0.016 (2)	−0.002 (2)
C12	0.044 (3)	0.059 (3)	0.063 (3)	−0.003 (2)	0.011 (2)	−0.004 (3)
C13	0.048 (3)	0.062 (3)	0.088 (5)	−0.008 (2)	0.030 (3)	0.003 (3)
C14	0.051 (3)	0.052 (3)	0.071 (4)	0.005 (2)	0.036 (3)	0.013 (3)
C15	0.059 (3)	0.050 (3)	0.049 (3)	0.013 (2)	0.028 (2)	0.014 (2)
C16	0.080 (4)	0.117 (5)	0.046 (3)	−0.026 (4)	0.015 (3)	−0.022 (3)
C17	0.068 (3)	0.083 (4)	0.030 (3)	0.007 (3)	0.006 (2)	0.005 (2)
C18	0.087 (4)	0.068 (4)	0.037 (3)	0.002 (3)	0.026 (3)	0.004 (2)
C19	0.127 (6)	0.090 (5)	0.110 (6)	0.039 (5)	0.038 (5)	0.022 (5)

### Geometric parameters (Å, °)

**Table:**

Cu1—O1	1.881 (3)	C4—H4	0.9300
Cu1—O2	1.887 (3)	C5—C6	1.360 (7)
Cu1—N1	1.905 (4)	C5—H5	0.9300
Cu1—N2	1.922 (4)	C6—H6	0.9300
Cl1—O6	1.394 (4)	C7—H7	0.9300
Cl1—O5	1.408 (4)	C8—H8A	0.9600
Cl1—O7'	1.410 (5)	C8—H8B	0.9600
Cl1—O5'	1.413 (5)	C8—H8C	0.9600
Cl1—O6'	1.418 (5)	C9—C14	1.403 (6)
Cl1—O7	1.421 (4)	C9—C10	1.410 (6)
Cl1—O8	1.424 (5)	C9—C15	1.432 (7)
Cl1—O8'	1.432 (5)	C10—C11	1.411 (6)
O1—C2	1.309 (5)	C11—C12	1.380 (6)
O2—C10	1.308 (5)	C12—C13	1.382 (7)
O3—C3	1.377 (5)	C12—H12	0.9300
O3—C8	1.415 (5)	C13—C14	1.357 (7)
O4—C11	1.378 (5)	C13—H13	0.9300
O4—C16	1.417 (6)	C14—H14	0.9300
O9—C19	1.362 (8)	C15—H15	0.9300
O9—H9A	0.8200	C16—H16A	0.9600
N1—C7	1.285 (6)	C16—H16B	0.9600
N1—C17	1.470 (6)	C16—H16C	0.9600
N2—C15	1.267 (6)	C17—C18	1.504 (7)
N2—C18	1.444 (6)	C17—H17A	0.9700
C1—C6	1.400 (7)	C17—H17B	0.9700
C1—C2	1.402 (6)	C18—H18A	0.9700
C1—C7	1.435 (6)	C18—H18B	0.9700
C2—C3	1.400 (6)	C19—H19A	0.9600
C3—C4	1.370 (6)	C19—H19B	0.9600
C4—C5	1.393 (7)	C19—H19C	0.9600
			
O1—Cu1—O2	86.23 (12)	O3—C8—H8B	109.5
O1—Cu1—N1	94.56 (15)	H8A—C8—H8B	109.5
O2—Cu1—N1	174.99 (15)	O3—C8—H8C	109.5
O1—Cu1—N2	174.73 (15)	H8A—C8—H8C	109.5
O2—Cu1—N2	94.09 (15)	H8B—C8—H8C	109.5
N1—Cu1—N2	85.58 (16)	C14—C9—C10	119.2 (4)
O6—Cl1—O5	113.8 (5)	C14—C9—C15	118.2 (4)
O7'—Cl1—O5'	129.6 (11)	C10—C9—C15	122.5 (4)
O7'—Cl1—O6'	111.9 (10)	O2—C10—C9	125.0 (4)
O5'—Cl1—O6'	88.9 (10)	O2—C10—C11	117.6 (4)
O6—Cl1—O7	112.0 (5)	C9—C10—C11	117.4 (4)
O5—Cl1—O7	99.0 (5)	O4—C11—C12	124.9 (4)
O6—Cl1—O8	112.6 (5)	O4—C11—C10	112.8 (4)
O5—Cl1—O8	104.2 (4)	C12—C11—C10	122.2 (4)
O7—Cl1—O8	114.3 (5)	C11—C12—C13	118.9 (5)
O7'—Cl1—O8'	100.0 (10)	C11—C12—H12	120.5
O5'—Cl1—O8'	129.9 (11)	C13—C12—H12	120.5
O6'—Cl1—O8'	76.9 (10)	C14—C13—C12	120.8 (5)
C2—O1—Cu1	126.7 (3)	C14—C13—H13	119.6
C10—O2—Cu1	126.5 (3)	C12—C13—H13	119.6
C3—O3—C8	117.9 (4)	C13—C14—C9	121.5 (4)
C11—O4—C16	117.0 (4)	C13—C14—H14	119.3
C19—O9—H9A	109.5	C9—C14—H14	119.3
C7—N1—C17	121.4 (4)	N2—C15—C9	125.7 (4)
C7—N1—Cu1	125.4 (3)	N2—C15—H15	117.2
C17—N1—Cu1	113.1 (3)	C9—C15—H15	117.2
C15—N2—C18	120.9 (4)	O4—C16—H16A	109.5
C15—N2—Cu1	126.2 (3)	O4—C16—H16B	109.5
C18—N2—Cu1	112.8 (3)	H16A—C16—H16B	109.5
C6—C1—C2	120.1 (4)	O4—C16—H16C	109.5
C6—C1—C7	117.7 (4)	H16A—C16—H16C	109.5
C2—C1—C7	122.2 (4)	H16B—C16—H16C	109.5
O1—C2—C3	117.7 (4)	N1—C17—C18	108.4 (4)
O1—C2—C1	125.0 (4)	N1—C17—H17A	110.0
C3—C2—C1	117.3 (4)	C18—C17—H17A	110.0
C4—C3—O3	125.3 (4)	N1—C17—H17B	110.0
C4—C3—C2	121.7 (4)	C18—C17—H17B	110.0
O3—C3—C2	113.0 (4)	H17A—C17—H17B	108.4
C3—C4—C5	120.4 (5)	N2—C18—C17	110.5 (4)
C3—C4—H4	119.8	N2—C18—H18A	109.5
C5—C4—H4	119.8	C17—C18—H18A	109.5
C6—C5—C4	119.0 (5)	N2—C18—H18B	109.5
C6—C5—H5	120.5	C17—C18—H18B	109.5
C4—C5—H5	120.5	H18A—C18—H18B	108.1
C5—C6—C1	121.4 (5)	O9—C19—H19A	109.5
C5—C6—H6	119.3	O9—C19—H19B	109.5
C1—C6—H6	119.3	H19A—C19—H19B	109.5
N1—C7—C1	126.0 (4)	O9—C19—H19C	109.5
N1—C7—H7	117.0	H19A—C19—H19C	109.5
C1—C7—H7	117.0	H19B—C19—H19C	109.5
O3—C8—H8A	109.5		
			
O2—Cu1—O1—C2	−175.3 (3)	C2—C1—C6—C5	1.2 (7)
N1—Cu1—O1—C2	−0.2 (3)	C7—C1—C6—C5	178.4 (4)
N2—Cu1—O1—C2	91.1 (16)	C17—N1—C7—C1	172.8 (4)
O1—Cu1—O2—C10	−174.8 (4)	Cu1—N1—C7—C1	−4.1 (7)
N1—Cu1—O2—C10	86.0 (17)	C6—C1—C7—N1	−176.8 (4)
N2—Cu1—O2—C10	0.0 (4)	C2—C1—C7—N1	0.3 (7)
O1—Cu1—N1—C7	3.7 (4)	Cu1—O2—C10—C9	1.9 (6)
O2—Cu1—N1—C7	102.6 (16)	Cu1—O2—C10—C11	−179.5 (3)
N2—Cu1—N1—C7	−171.1 (4)	C14—C9—C10—O2	178.3 (4)
O1—Cu1—N1—C17	−173.5 (3)	C15—C9—C10—O2	−2.3 (7)
O2—Cu1—N1—C17	−74.6 (17)	C14—C9—C10—C11	−0.4 (6)
N2—Cu1—N1—C17	11.8 (3)	C15—C9—C10—C11	179.1 (4)
O1—Cu1—N2—C15	91.5 (16)	C16—O4—C11—C12	−3.9 (7)
O2—Cu1—N2—C15	−1.8 (4)	C16—O4—C11—C10	178.3 (4)
N1—Cu1—N2—C15	−176.8 (4)	O2—C10—C11—O4	0.1 (6)
O1—Cu1—N2—C18	−83.8 (16)	C9—C10—C11—O4	178.8 (4)
O2—Cu1—N2—C18	−177.1 (3)	O2—C10—C11—C12	−177.8 (4)
N1—Cu1—N2—C18	7.9 (3)	C9—C10—C11—C12	1.0 (7)
Cu1—O1—C2—C3	176.9 (3)	O4—C11—C12—C13	−178.4 (4)
Cu1—O1—C2—C1	−3.1 (6)	C10—C11—C12—C13	−0.8 (7)
C6—C1—C2—O1	−179.4 (4)	C11—C12—C13—C14	0.0 (8)
C7—C1—C2—O1	3.6 (7)	C12—C13—C14—C9	0.6 (8)
C6—C1—C2—C3	0.7 (6)	C10—C9—C14—C13	−0.4 (7)
C7—C1—C2—C3	−176.4 (4)	C15—C9—C14—C13	−179.9 (4)
C8—O3—C3—C4	6.6 (7)	C18—N2—C15—C9	176.8 (4)
C8—O3—C3—C2	−172.6 (4)	Cu1—N2—C15—C9	1.9 (7)
O1—C2—C3—C4	178.1 (4)	C14—C9—C15—N2	179.7 (4)
C1—C2—C3—C4	−1.9 (6)	C10—C9—C15—N2	0.2 (7)
O1—C2—C3—O3	−2.7 (6)	C7—N1—C17—C18	154.9 (4)
C1—C2—C3—O3	177.3 (4)	Cu1—N1—C17—C18	−27.8 (5)
O3—C3—C4—C5	−177.8 (4)	C15—N2—C18—C17	159.2 (4)
C2—C3—C4—C5	1.3 (7)	Cu1—N2—C18—C17	−25.3 (5)
C3—C4—C5—C6	0.6 (7)	N1—C17—C18—N2	33.7 (6)
C4—C5—C6—C1	−1.8 (7)		

### Hydrogen-bond geometry (Å, °)

**Table:**

*D*—H···*A*	*D*—H	H···*A*	*D*···*A*	*D*—H···*A*
O9—H9A···O7^i^	0.82	2.11	2.912 (8)	164
O9—H9A···O5'^i^	0.82	1.99	2.761 (13)	156
O9—H9A···O5'^i^	0.82	1.99	2.761 (13)	156
O9—H9A···O7^i^	0.82	2.11	2.912 (8)	164

Symmetry codes: (i) −*x*+3/2, *y*−1/2, −*z*+3/2.
